# Stunting, underweight and thinness in internationally adopted children: prevalence and associated factors in a large cohort study

**DOI:** 10.1007/s00431-026-07152-6

**Published:** 2026-06-26

**Authors:** Nicolo’ Monti, Maryia Kulak, Catiuscia Lisi, Yekatsiaryna Rakevich, Matteo Manca, Elisabetta Venturini, Leila Bianchi, Luisa Galli, Elena Chiappini

**Affiliations:** 1 Postgraduate School of Pediatrics, Department of Health Sciences, University of Florence, Florence, Italy; 2 Department of Health Sciences, University of Florence, Florence, Italy; 3 University of Florence, Florence, Italy; 4 Infectious Diseases Unit, Meyer Children’s Hospital IRCCS, Florence, 50139 Italy

**Keywords:** International adopted children, Stunting, Underweight, Thinness, Institutionalized children, Fetal alcohol spectrum disorder, Malnutrition, Growth impairment, Vitamin D deficiency, Parasitic infections

## Abstract

Internationally adopted children (IAC) are at high risk of growth impairment due to early-life deprivation, institutional care, and prenatal exposures. Data on determinants of stunting and other forms of malnutrition in this population remain limited. To evaluate the prevalence of stunting, underweight, and thinness, and to identify factors associated with these conditions in a large cohort of internationally adopted children (IAC), we conducted a large cohort study including 1,955 IAC referred to a tertiary pediatric center in Italy between 2015 and 2025. The cohort mainly included children aged 5–9 years (52.2%) and 1–4 years (33.8%), while only a minority were younger than 1 year or older than 14 years. Growth parameters were assessed using WHO standards. Stunting (HAZ < − 2 SD) and thinness (BMIZ < − 2 SD) were assessed in children aged 1 month to 18 years, while underweight (WAZ < − 2 SD) was evaluated in children younger than 10 years. Demographic, clinical, and laboratory variables were analyzed using a population-averaged generalized estimating equations (GEE) model, followed by univariate and multivariable logistic regression analyses for specific growth outcomes. An interaction analysis between low ferritin and abnormal TSH was also performed. A total of 1,955 internationally adopted children (IAC) were included in the analysis. Stunting was observed in 12.2% of children, underweight in 12.7%, and thinness in 5.6%. In the GEE model evaluating overall growth impairment (stunting, underweight and thinness), independent risk factors included anemia, younger age (1–4 years), Asian origin, and fetal alcohol spectrum disorder (FASD), with FASD showing the strongest association (OR 4.77, 95% CI 3.32–6.86; *p* < 0.001). In outcome specific univariate analyses, independent predictors of stunting included age 1–4 years (OR 3.09), Asian origin (OR 1.48), anemia (OR 2.30), FASD (OR 4.26), and abnormal thyroid-stimulating hormone (TSH) levels (OR 1.83). Anemia was significantly associated with parasitic infections (*p* = 0.05), supporting a potential contribution of infectious burden to nutritional impairment. Among children with available ferritin and thyroid data, hypoferritinemia was significantly associated with abnormal TSH levels (*p* = 0.024); however, this association was not confirmed in univariate logistic regression.

*Conclusions*: A high prevalence of stunting persists among IAC at first presentation to our center. Both prenatal factors, particularly FASD, and postnatal conditions contribute to malnutrition. Early comprehensive screening, including nutritional, infectious, and endocrine assessment, is essential to identify at-risk children and guide targeted interventions.

**Table:** 

**What is known- What is new:** *The IAC are at high risk of growth impairment due to early-life deprivation, institutional care, and prenatal exposures, with stunting frequently reported at arrival. In this large cohort of IAC, stunting affected 12.2% of children and was independently associated with FASD, young age, Asian origin, and anemia, highlighting the combined role of prenatal and postnatal factors and the need for comprehensive early screening*.

**Graphical Abstract:**

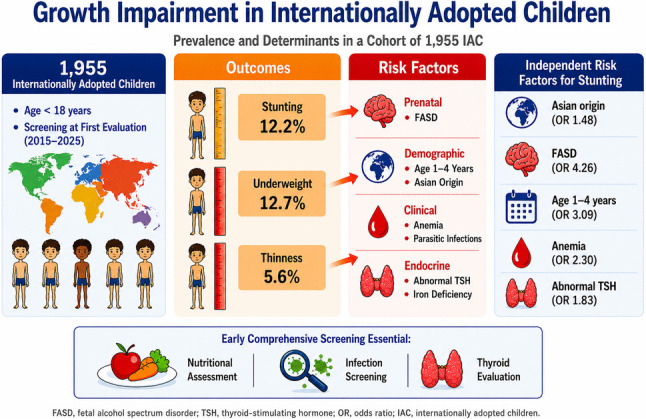

**Supplementary Information:**

The online version contains supplementary material available at 10.1007/s00431-026-07152-6.

## Background

Child malnutrition remains one of the most significant threats to child survival, growth, and development worldwide [1]. Stunting, defined as a height-for-age Z-score (HAZ) below − 2 standard deviations (SD) according to World Health Organization (WHO) Child Growth Standards, is widely recognized as a marker of chronic undernutrition and cumulative early-life adversity [2–4]. It reflects prolonged exposure to nutritional deficiencies, recurrent infections, inadequate caregiving, and socio-environmental deprivation [2, 5]. Stunting is associated with increased childhood morbidity and mortality, impaired neurocognitive development, reduced educational attainment, and long-term adverse cardiometabolic outcomes in adulthood [1, 6–9]. Weight-for-age Z-score (WAZ) below − 2 SD, in children under 10 years, identifies underweight; a composite indicator reflecting both chronic and acute undernutrition. In contrast, BMI-for-age Z-score (BMIZ) below − 2 SD identifies thinness, which reflects low body mass relative to height and therefore provides a more specific indicator of body proportionality, particularly in school-aged children and adolescents [10].

Internationally adopted children (IAC) raised in institutional settings represent one of the highest-risk groups for stunting [11]. Institutional care is frequently characterized by “structural neglect”, including frequent caregiver turnover, limited individualized feeding, and reduced psychosocial stimulation [11]. These conditions can adversely affect growth through both nutritional pathways and stress-related neuroendocrine mechanisms [11]. In a systematic review including 25 studies, children living in institutionalized care frequently showed stunting and underweight, with HAZ and WAZ often below international standards and worse than those observed in community comparison groups [11]. Prolonged residence in institutional settings has been associated with greater delays in both height and weight gain, whereas placement into family-based environments, including foster care or adoptive families, often results in substantial catch-up growth [12, 13].

Nevertheless, early deprivation during critical developmental windows may produce persistent effects on growth trajectories and metabolic regulation [13]. Catch-up growth following early deprivation is biologically adaptive but may also carry long-term consequences as earlier pubertal onset, altered hypothalamic–pituitary–adrenal axis regulation, thyroid dysfunction, and increased cardiometabolic risk later in life [13–15].

In addition to postnatal environmental deprivation, prenatal factors may also contribute substantially to growth impairment among children raised in institutional settings [16]. Among these, fetal alcohol spectrum disorders (FASD) represent a major but often under-recognized determinant of impaired growth in IAC [13, 16, 17]. FASD represent a continuum of conditions caused by prenatal alcohol exposure, including fetal alcohol syndrome (FAS), partial fetal alcohol syndrome (pFAS), and alcohol-related neurodevelopmental disorder (ARND/ND-PAE) [16, 17]. These conditions differ in the combination and severity of clinical features involving growth, morphology, and neurodevelopment. The FAS is the most severe form and is characterized by the presence of all three domains: growth impairment (prenatal and/or postnatal), characteristic facial dysmorphisms, and central nervous system abnormalities [16, 17]. The pFAS presents with a partial expression of these features, typically including characteristic facial anomalies and neurodevelopmental impairment, with or without growth deficits [16, 17]. In contrast, ARND/ND-PAE is defined by neurodevelopmental abnormalities in the absence of the typical facial features and growth restriction, requiring confirmed prenatal alcohol exposure [16, 17].

The primary aim of the study was to assess the prevalence of stunting and to identify clinical, laboratory, and epidemiological factors associated with growth impairment in a large cohort of internationally adopted children referred for medical screening at an Italian tertiary pediatric center over a ten-year period. Secondary aims were to evaluate the prevalence of underweight and thinness and to explore potential risk factors associated with these conditions. Furthermore, based on previous evidence, we evaluated the association between iron deficiency and thyroid dysfunction in our cohort [18]. Considering the high prevalence of parasitic infections in internationally adopted children and their potential impact on intestinal absorption, we also investigated the relationship between parasitic infections, anemia, and hypoferritinemia as a potential indicator of malabsorption [19]. Understanding the determinants of growth impairment in this population may provide important insights for early clinical management, targeted nutritional interventions, and long-term health monitoring.

## Material and method

### Study design and population

This retrospective cohort study was conducted at Meyer Children’s University Hospital IRCCS, a tertiary pediatric referral center in Florence, Italy, between January 2015 and February 2025. All enrolled children underwent a standardized screening at the first clinical evaluation, developed in accordance with the American Academy of Pediatrics (AAP) recommendations [20].

All internationally adopted children (IAC) aged < 18 years, originating from foreign countries, who were consecutively referred to our center for post-adoption medical evaluation during the study period were enrolled. Exclusion criteria were adoption from Italy and/or age ≥ 18 years. Clinical and laboratory data were collected and entered into a dedicated electronic database [21].

Written informed consent was obtained from the adoptive parents or legal guardians of all enrolled children. The study was approved by the Ethics Committee for Human Investigation of Meyer Children’s University Hospital (Protocol No.248/2024), Italy.

### Data collection and clinical assessment

#### Data collection

Collected variables included:

- **Demographics and background:** sex, age, country of origin, comorbidities or immunodeficiencies, and history of international travel.
- **Clinical presentation:** signs and symptoms at initial evaluation. Particular attention was paid to clinical features suggestive of FASD [16, 17]. Height (or length, when appropriate) and weight were measured according to WHO recommendations [22]. HAZ, BMIZ, and WAZ were calculated using the WHO AnthroPlus software [23].
- **Laboratory data:** including hemoglobin levels, white blood cell count, absolute eosinophil count, thyroid function (TSH) , 25-OH-D3 levels, ferritin, Quantiferon and tuberculin skin test.
- **Microbiological results:** IgG-TES-ELISA and IgG-TES-Western Blot for *Toxocara spp.*; stool parasitology; and serology for *Strongyloides stercoralis*, *Schistosoma mansoni*, *Taenia solium*, and *Trypanosoma cruzi* in children from endemic areas.

### Definitions

According to WHO criteria [22–24], stunting was defined as HAZ < − 2 standard deviations (SD), Underweight was defined as WAZ < − 2 SD; according to WHO, WAZ reference data are not available beyond 10 years. [23]. Thiness was defined as BMIZ < − 2 SD and may result from stunting, wasting, or both [23].

Children presenting with facial dysmorphic features and/or behavioral, cognitive, or intellectual abnormalities suggestive of FASD were evaluated according to the diagnostic criteria [16, 17]. Clinical assessment specifically included evaluation of the characteristic facial features associated with FASD, namely short palpebral fissures, smooth philtrum, and thin vermilion border of the upper lip, together with assessment of growth parameters (weight, height, head circumference/microcephaly) and neurodevelopmental abnormalities suggestive of central nervous system involvement. Prenatal alcohol exposure was assessed through review of available maternal, obstetric, and perinatal history documented in the medical records [16, 17]. Within the FASD spectrum, cases were classified as FAS or non-FAS disorders (pFAS and ARND/ND-PAE) according to established diagnostic criteria [16, 17]. All children with suspected FASD were referred to the clinical genetics team at our hospital for comprehensive evaluation. The genetic assessment aimed to exclude alternative genetic syndromes or other etiologies that could explain the child’s dysmorphic, neurodevelopmental, or behavioral phenotype, in line with recommended differential diagnostic work-up for FASD. When indicated, genetic testing was performed at the discretion of the clinical geneticist. Final diagnostic classification was established after this multidisciplinary evaluation, and the results of the genetic investigations together with confirmation of FASD diagnosis were communicated to the family and to our clinical unit.

Vitamin D deficiency was defined as serum 25(OH)D < 20.0 ng/mL [25]. The reference range for TSH was defined within 0.4–3.9 mIU/L [26]. Anemia was defined as hemoglobin level < 11 g/dL [27]. Serum ferritin levels were considered within the normal range when comprised between 15 and 300 ng/mL [27]. According to the WHO and the International Consensus Classification of eosinophilic disorders, eosinophilia was classified into three groups: mild (500–1,500 cells/µL), moderate (1,500–5,000 cells/µL), and severe (> 5,000 cells/µL) [28].

According to current international terminology, tuberculosis infection (TBI) ranges from non-infectious states, through subclinical forms (asymptomatic or unrecognized by the individual), which may be either non-infectious or transmissible, to clinically manifest active disease [29].

Parasitic infection was defined as the presence of at least one laboratory test positive for a pathogenic parasite, including either positive serology for parasite-specific antibodies or a positive stool examination (microscopy or antigen detection) identifying parasitic elements such as cysts, trophozoites, larvae, or eggs [30].

### Laboratory and microbiological assessment

At first evaluation, all children underwent venipuncture for laboratory testing, including: complete blood count with differential, serum 25-hydroxyvitamin D [25(OH)D] and thyroid-stimulating hormone. Serum 25(OH)D levels were measured using a chemiluminescent enzyme-labeled immunometric assay (Immulite 2000 Systems Analyzer, Siemens, Gwynedd, UK). Serum TSH levels were determined using a third-generation immunometric assay (Immulite 2000, DPC Diagnostic Products Corporation, Los Angeles, CA, USA).

Serological tests were carried out in children to detect specific IgG antibodies against *Toxocara spp.*, *Strongyloides stercoralis*, *Schistosoma mansoni*, *Taenia solium*, and *Trypanosoma cruzi* [31]. For the diagnosis of *Toxocara* spp. infection, an ELISA test (CHORUS Toxocara IgG, Biomedical Diagnostics, Antwerp, Belgium) was used. In cases where serological results were doubtful or inconclusive, further confirmation was obtained by Western Blot (Toxocara spp. WB, LDBIO Diagnostic, France). For the diagnosis of tuberculosis, an interferon-γ release assay (IGRA) was performed. The test was considered positive when the INF-γ level in plasma stimulated with specific mycobacterial antigens exceeded by at least 0.35 IU/ml the amount measured in the negative control sample. The tuberculin skin test (TST, Mantoux method) was performed by intradermal injection of 0.1 mL of purified protein derivative (PPD) into the volar surface of the forearm. The test was read 48–72 h after administration by measuring the transverse diameter of induration in millimeters, according to international pediatric tuberculosis screening recommendations [32]. Microscopic analysis for enteric protozoan cysts and/or trophozoites was performed according to the standardized procedures [31]. Antigen detection for *Cryptosporidium* and *Giardia* was performed using an immunochromatographic assay (Stick Crypto/Giardia; Operon®, Zaragoza, Spain) according to the manufacturer’s instructions [31].

### Statistical analysis

Data are presented as absolute numbers and percentages for categorical variables, and continuous variables are reported as median and interquartile range (IQR). Comparisons between groups were performed using the Chi-square test or Fisher’s exact test for categorical variables, as appropriate. Univariate logistic regression analyses were performed to evaluate the association between potential risk factors and growth impairment outcomes, including stunting, underweight, and thinness. To evaluate factors associated with stunting, underweight and thinness, we used a two-step analytical strategy. Since stunting, underweight, and thinness represent related anthropometric manifestations of impaired growth within the same individual, we first performed a population-averaged generalized estimating equations (GEE) analysis to jointly model these correlated binary outcomes (growth impairment *vs* not growth impairment). For this purpose, the dataset was restructured in long format, with each child contributing one observation for each growth outcome (stunting, underweight, and thinness). A binomial distribution with logit link function was specified, using an exchangeable working correlation structure and robust standard errors clustered by individual participant to account for within-subject correlation. Covariates included sex, age, anemia, TSH, 25(OH)D , continent of origin, and fetal alcohol spectrum disorder. This approach enabled identification of variables independently associated with the overall burden of growth impairment (stunting, underweight and thinness), rather than with a single anthropometric indicator alone. To further explore which specific outcome (stunting, underweight and thinness), predominantly drove the associations observed in the combined GEE model, we subsequently performed separate univariate logistic regression analyses for each outcome. This step-down exploratory analysis was undertaken to disentangle the relative contribution of stunting, underweight and thinness to the overall associations identified in the primary model. The following variables were analyzed in the univariate models: sex, age class, continent of origin, time since arrival in Italy, presence or suspected fetal alcohol spectrum disorders, anemia, ferritin levels, eosinophilia, vitamin D status, and tuberculosis or parasitic infections. To explore the potential interaction between iron deficiency and thyroid dysfunction in relation to stunting, we performed a logistic regression analysis including low ferritin levels, abnormal TSH values, and their interaction term (low ferritin × abnormal TSH) among children with available ferritin and TSH measurements. Results from GEE, univariate and logistic regression analyses are presented as odds ratios (ORs) with 95% confidence intervals (95% CIs). A two-sided p value < 0.05 was considered statistically significant. All analyses were performed using Stata (StataCorp, College Station, TX, USA).

## Results

**Fig. 1:**
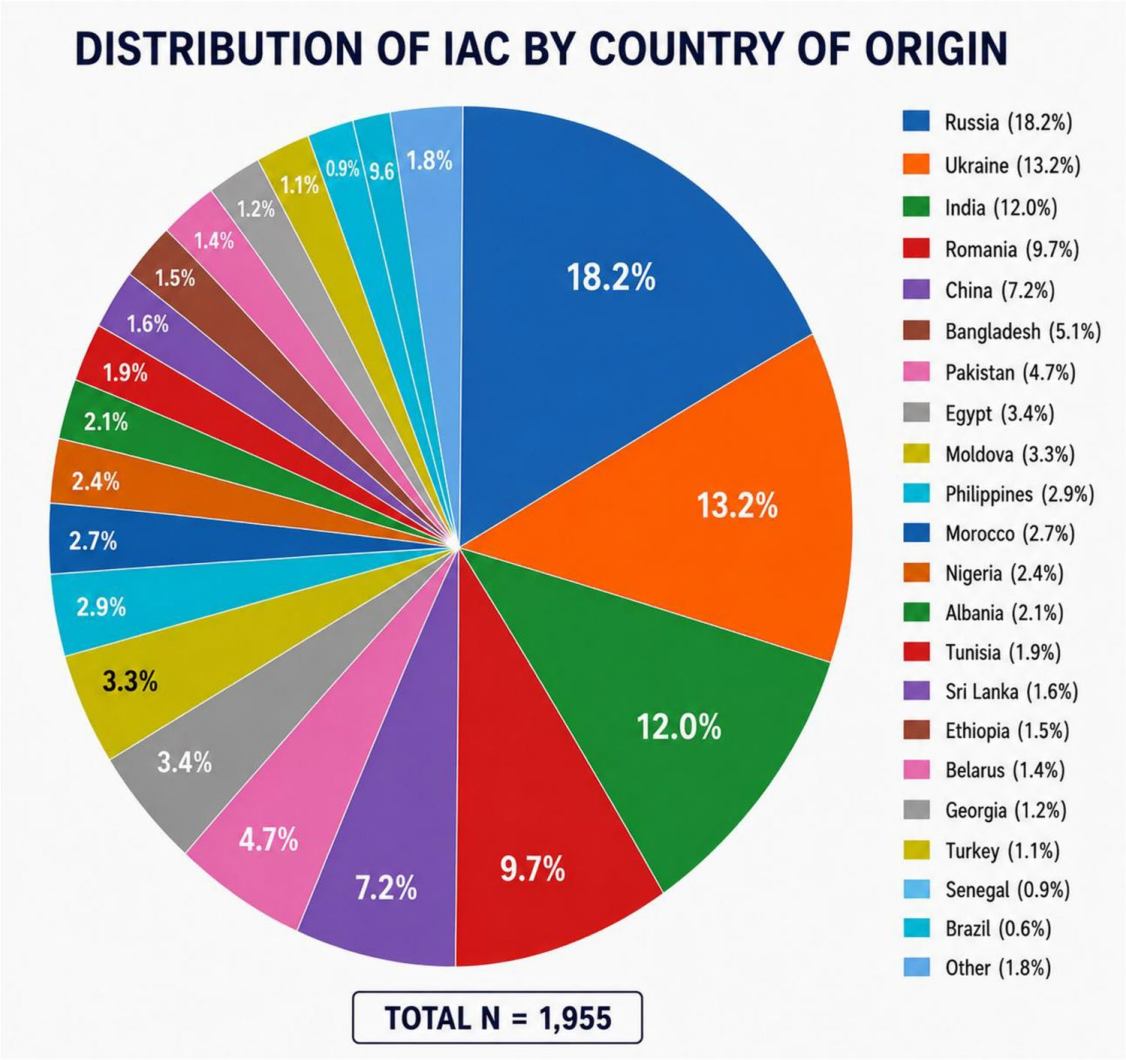
Geographical distribution of internationally adopted children by country of origin. Pie chart showing the distribution of the 1,955 internationally adopted children (IAC) included in the study according to country of origin at the time of first post-adoption medical evaluation. Percentages represent the proportion of children originating from each country. Countries with very low representation were grouped under “Other”

**Fig. 2:**
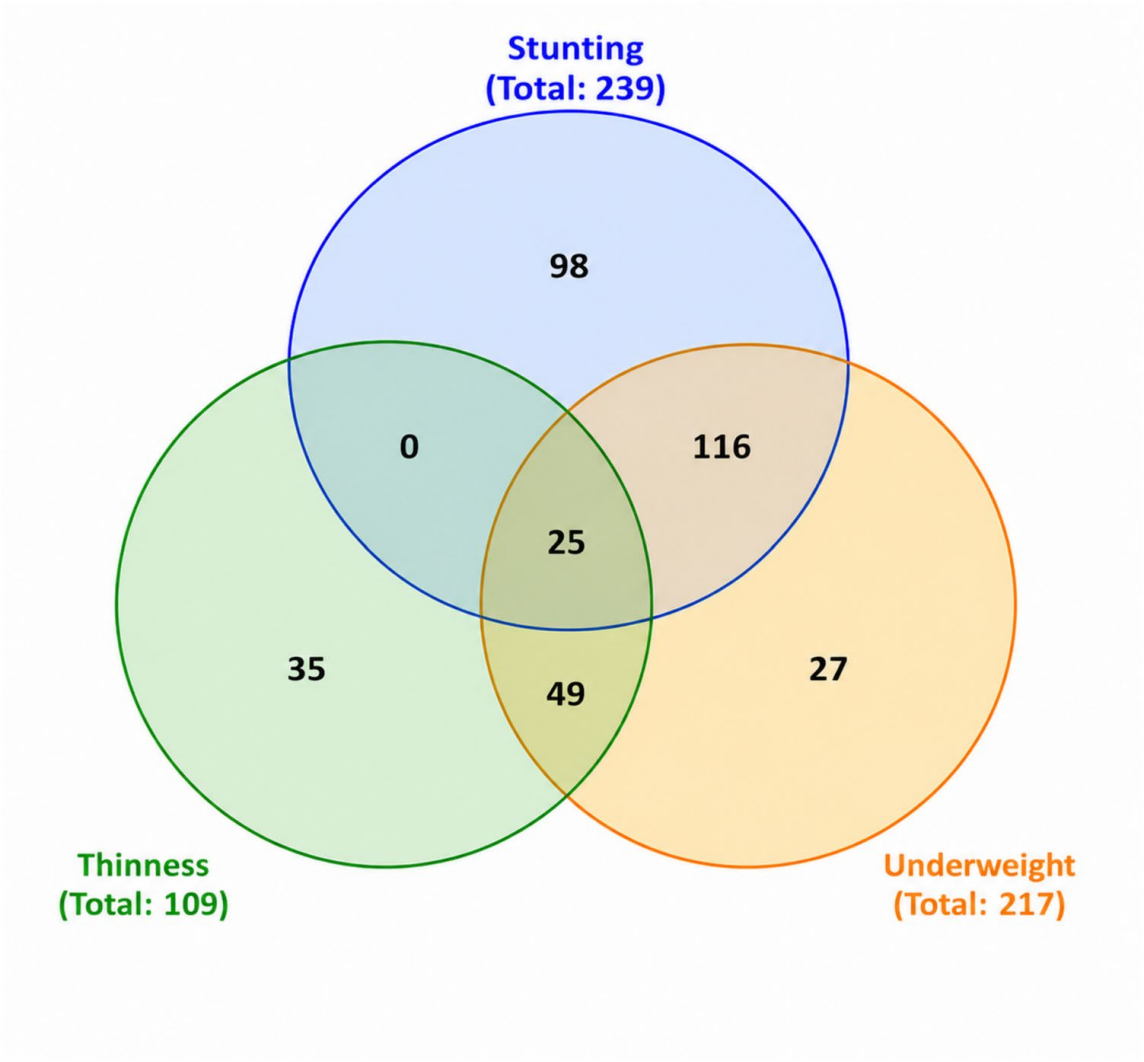
Overlap of growth impairment outcomes in internationally adopted children. Venn diagram illustrating the overlap between the three growth impairment outcomes evaluated in the study: stunting (height-for-age Z-score < − 2 SD), underweight (weight-for-age Z-score < − 2 SD), and thinness (BMI-for-age Z-score < − 2 SD) among internationally adopted children. Numbers indicate the absolute number of children presenting with each isolated condition or combinations of conditions

A total of 1,955 internationally adopted children were included in the analysis for height-for-age Z-score and BMI-for-age Z-score (Tables 1 and 2). Weight-for-age Z-score was evaluated in 1,709 children (Table 3). The characteristics of the entire IAC cohort included in the study was summarized in supplementary material Table S1 and Fig. 1. Only 12 children (0.5%) originated from foster/family care settings, all from South America, whereas most children (99.5%) came from institutional care. Stunting was observed in 239 (12.2%), thinness in 109 (5.6%) and underweight in 217 (12.7%) (Tables 1–3). The median HAZ was − 0.56 (IQR − 1.34 to 0.27; N = 1955), the median BMIZ was − 0.10 (IQR − 0.92 to 0.72; N = 1955) and the median WAZ was − 0.44 (IQR − 1.25 to 0.45; N = 1709). A total of 160 children (8.4%) presented with an isolated condition among stunting, underweight, and thinness; 165 children (8.4%) presented with two of these conditions, and 25 children (1.3%) presented with all three. The distribution of children with one or more growth impairments (stunting, underweight, and thinness) is shown in Fig. 2. Stunting prevalence was significantly lower in children residing in Italy for > 90 days compared with < 90 days (*p* = 0.044), results not confirmed in univariate analysis, likewise for underweight and thinness (Tables 1–3, Supplementary material table S2-S4).

**Table 1:** Comparison of internationally adopted children with stunting (HAZ < –2DS) versus normal growth

Total of internationally adopted children (*n* = 1955)	Internationally adopted children with stunting (HAZ < −2DS) *n* = 239	Internationally adopted children without stunting (HAZ ≥ −2DS) *n* = 1716	*p*-value
Gender			
Male	144 (60.4%)	1042 (60.7%)	*P* = 0.932
Female	95 (39.5%)	674 (39.3%)	
Continent of origin			
Europe	105 (43.8%)	679 (39.6%)	
Asia	76 (32%)	342 (19.9%)	***P*** **= 0.02**
Africa	25 (10.4%)	273 (15.9%)	***P*** **= 0.02**
America	31 (12.9%)	416 (24.3%)	***P*** **< 0.001**
Unknown	2 (0.83%)	6 (0.35%)	*P*=0.33
Age in years			
< 1 year	2 (0.83%)	27 (1.6%)	*P* = 0.92
1–4 years	144 (60.4%)	516 (30%)	***P*** **< 0.001**
5–9 years	75 (31.3%)	946 (55.1%)	
10–14 years	17 (7.1%)	190 (11.0%)	*P* = 0.66
≥ 15 years	1 (0.41%)	37 (2.2%)	*P* = 0.27
Days since arrival in Italy, [median, (IQR)	[61, (39–107)]	[76, (46–125)]	
1–90 days	155 (65%)	1017 (59.3%)	*P* = 0.099
> 90 days	84 (35%)	699 (40.7%)	
Eosinophilia			
No	197 (82.5%)	1411 (82.2%)	*P* = 0.914
Yes	42 (17.5%)	305 (17.8%)	
Mild	35 (14.6%)	275 (16%)	
Moderate	6 (2.5%)	30 (1.7%)	
Severe	1 (0.41%)	0 (0%)	
Hb			
< 11 g/dl	24 (10.4%)	85 (5.0%)	***P*** ** = 0.001**
≥ 11 g/dl	215 (89.6%)	1631 (95%)	
Ferritin			
< 15ng/ml	20 (8.4%)	100 (5.8%)	*P* = 0.069
15–300 ng/ml	58 (24.6%)	490 (28.5%)	
Not performed	161 (67%)	1126 (65.7%)	
Vitamin D			
< 20 ng/ml	72 (30%)	696 (40.6%)	***P* = 0.0022**
≥ 20 ng/ml	160 (67%)	988(57.6%)	
Not performed	7 (3%)	32 (1.9%)	
TSH range [0,4–3,9 mIU/L]			
In range	183 (76.7%)	1478 (86.1%)	***P*** ** = 0.001**
Not in range	44 (18.3%)	194 (11.3%)	
Not performed	12 (5%)	44 (2.6%)	
Coinfection parassities			
No	163 (68.3%)	1106 (64.4%)	*P* = 0.22
Yes	75 (31.3%)	605 (35.3%)	
Not performed	1 (0.4%)	5 (0.3%)	
TBC			
No	208 (87%)	1547 (90.1%)	*P* = 0.17
Yes	30 (12.5%)	166 (9.6%)	
Latent	29 (12%)	157 (9.2%)	
Active	1 (0.4%)	9 (0.52%)	
Not performed	1 (0.4%)	3 (0.2%)	
Fetal-Alcohol Spectrum Disorder			
Negative	205 (85.8%)	1655 (96.4%)	***P*** ** < 0.001**
FASD	34 (14.1%)	61 (3.5%)	
FAS	11 (4.6%)	15 (0.9%)	
pFAS, ARND/ND-PAE	23 (9.6%)	46 (2.7%)	

*HAZ* height-for-age Z-score; *DS*, standard deviations; I*QR* interquartile range; *Hb* hemoglobin; *TSH* thyroid-stimulating hormone; *TBC* tuberculosis; *FASD* fetal alcohol spectrum disorder; *FAS* fetal alcohol syndrome; *pFAS* partial fetal alcohol syndrome; *ARND* alcohol-related neurodevelopmental disorder; *ND-PAE* neurodevelopmental disorder associated with prenatal alcohol exposure

**Table 2:** Clinical and demographic characteristics of internationally adopted children with and without thinness (BMIZ < –2DS)

Total of children (N = 1955)	Internationally adopted children with thinness (BMIZ < −2 DS) (*n* = 109)	Internationally adopted children without thinness (BMIZ ≥ −2 DS) (*n* = 1846)	*p*-value
Gender			
Male	74 (67.9%)	1112 (60.3%)	*P* = 0.26
Female	35 (32.1%)	734 (39.7%)	
Continent of origin			
Europe	53 (48.6%)	731 (39.6%)	
Asia	42 (38.5%)	376 (20.4%)	***P*** ** = 0.01**
Africa	10 (9.2%)	288 (15.6%)	***P*** ** = 0.03**
America	4 (3.7%)	443 (24.0%)	***P*** ** < 0.001**
Unknown	0 (0%)	8 (0.4%)	*P* = 0.44
Age in years			
< 1 year	4 (3.7%)	25 (1.3%)	***P*** ** = 0.01**
1–4 years	49 (45.0%)	611 (33%)	***P*** ** < 0.001**
5–9 years	45 (41.3%)	976 (52.9%)	
10–14 years	11 (10%)	196 (10.7%)	*P* = 0.57
> 15 years	0 (0%)	38 (2.1%)	*P* = 0.18
Days since arrival in Italy [median (IQR)]	[72, (45-125)]	[75, (45-124)]	
1–90 days	60 (55%)	1112 (60.2%)	*P* = 0.28
> 90 days	49 (45%)	734 (39.8%)	
Eosinophilia			
No	89 (81.7%)	1519 (82.3%)	*P* = 0.866
Yes	20 (18.3%)	327 (17.7%)	
Hb			
< 12 g/dl	6 (5.5%)	104 (5.6%)	*P* = 0.95
≥ 12 g/dl	103 (94.5%)	1742 (94.4%)	
Ferritin			
< 15ng/ml	9 (8.3%)	111 (6.0%)	*P* = 0.18
15–300 ng/ml	25 (23%)	523 (28.5%)	
Not performed	75 (68.8%)	1202 (65.4%)	
Vitamin D			
< 20 ng/ml	35 (32.1%)	733 (39.7%)	*P* = 0.16
≥ 20 ng/ml	73 (67%)	1075 (58.2%)	
Not performed	1 (0.9%)	38 (2.1%)	
TSH range [0,4-3,9 mIU/L]			
In range	89 (81.7%)	1572 (85.2%)	*P* = 0.27
Not in range	15 (13.8%)	223 (12%)	
Not performed	5 (4.6%)	51 (2.8%)	
Coinfection parassities			
No	74 (67.9%)	1195 (64.7%)	*P* = 0.27
Yes	35 (32.1%)	645 (35.3%)	
TBC			
No	97 (89%)	1658 (89.8%)	*P* = 0.96
Yes	11 (11%)	185 (10.2%)	
Latent	11 (9.2%)	175 (9.5%)	
Active	1 (0.8%)	9 (0.5%)	
Not performed	1 (0.8%)	3 (0.2%)	
Fetal-Alcohol Spectrum			
Disorder			***P*** **= 0.0015**
Negative	96 (88%)	1764 (95.6%)	
FASD	13 (12%)	82 (4.4%)	
FAS	7 (5.9%)	19 (1.0%)	
pFAS, ARND/ND-PAE	6 (5.0%)	63 (3.4%)	

*BMIZ* refers to body mass index-for-age Z-score; *DS* standard deviations; IQR, interquartile range; *Hb* hemoglobin; *TSH* thyroid-stimulating hormone; *TBC* tuberculosis; *FASD* fetal alcohol spectrum disorder; *FAS* fetal alcohol syndrome; *pFAS* partial fetal alcohol syndrome; *ARND* alcohol-related neurodevelopmental disorder; *ND-PAE* neurodevelopmental disorder associated with prenatal alcohol exposure

**Table 3:** Comparison of internationally adopted children with underweight (WAZ < –2DS) versus normal weight-for-age

Total of children (*N* = 1709)	Internationally adopted children with wasting (WAZ < −2DS) (*n* = 217)	Internationally adopted children without wasting (WAZ > −2DS) (*n* = 1492)	*p*-value
Gender			
Male	135 (61.9%)	907 (60.8%)	*P* = 0.75
Female	82 (38.1%)	585 (39.2%)	
Continent of origin			
Europe	107 (49.5%)	573 (38.4%)	
Asia	78 (35.8%)	306 (20.5%)	***P*** ** = 0.05**
Africa	17 (7.8%)	251 (16.8%)	***P*** ** < 0.001**
America	13 (6.0%)	357 (23.9%)	***P*** ** < 0.001**
Unknown	2 (0.9%)	5 (0.3%)	***P*** ** = 0.35**
Age in years			
< 1 year	5 (2.3%)	24 (1.6%)	*P* = 0.09
1–4 years	126 (57.8%)	533 (35.7%)	***P*** ** < 0.001**
5–9 years	86 (39.9%)	935 (62.6%)	
10–14 years	Not included	Not included	
> 15 years	Not included	Not included	
Days since arrival in Italy, [median, (IQR)	[63, (39–102)]	[73, (44–116)]	
1–90 days	146 (67.3%)	930 (62.3%)	*P* = 0.158
> 90 days	71 (32.7%)	562 (37.7%)	
Eosinophilia			
No	174 (80.3%)	1232 (82.6%)	*P* = 0.40
Yes	43 (19.7%)	260 (17.4%)	
Mild	34 (15.6%)	233 (15.6%)	
Moderate	8 (3.7%)	27 (1.8%)	
Severe	1 (0.5%)	0 (0.0%)	
Hb			
< 11 g/dl	17 (7.8%)	88 (5.9%)	*P* = 0.266
≥ 11 g/dl	200 (92.2%)	1405 (94.1%)	
Ferritin			
<15ng/ml	18 (8.3%)	88 (5.9%)	*P* = 0.12
15–300 ng/ml	54 (25.2%)	422 (28.2%)	
Not performed	145 (66.5%)	982 (65.9%)	
Vitamin D			
< 20 ng/ml	59 (27.1%)	587 (39.4%)	***P*** ** = 0.00059**
≥ 20 ng/ml	153 (70.2%)	876 (58.8%)	
Not performed	6 (2.8%)	28 (1.9%)	
TSH range [0.4–3.9 mIU/l]			
In range	172 (79.4%)	1266 (84.8%)	*P* = 0.29
Not in range	32 (14.6%)	188 (12.6%)	
Not performed	13 (6%)	39 (2.6%)	
Coinfection parassities			
No	150 (69.3%)	957 (64.1%)	*P* = 0.12
Yes	66 (30.3%)	531 (35.6%)	
Not performed	1 (0.5%)	5 (0.3%)	
TBC			
No	187 (86.2%)	1360 (91.1%)	***P*** ** = 0.048**
Yes	29 (13.4%)	130 (8.8%)	
Latent	28 (13%)	124 (8.4%)	
Active	1 (0.4%)	6 (0.4%)	
Not performed	1 (0.4%)	1 (0.1%)	
Fetal-Alcohol Spectrum Disorder			
Negative	175 (80.7%)	1450 (97.1%)	***P*** ** < 0.001**
FASD	42 (19.3%)	43 (2.9%)	
FAS	16 (7.4%)	9 (0.6%)	
pFAS, ARND/ND-PAE	26 (11, 9%)	34 (2.3%)	

*WAZ* refers to weight-for-age Z-score < −2; *DS* standard deviations; *IQR* interquartile range; *Hb* hemoglobin; *TSH*, thyroid-stimulating hormone; *TBC* tuberculosis; *FASD* fetal alcohol spectrum disorder; *FAS*, fetal alcohol syndrome; *pFAS* partial fetal alcohol syndrome; *ARND* alcohol-related neurodevelopmental disorder; *ND-PAE* neurodevelopmental disorder associated with prenatal alcohol exposure

### Risk factor for stunting, underweight and thinness

In the adjusted GEE model, anemia, younger age (1–4 years), Asian origin, and FASD were independently associated with higher odds of growth impairment (stunting, underweight and thinness), with FASD showing the strongest association (OR 4.77, 95% CI 3.32–6.86; p < 0.001) (Table 4). In contrast, low levels of vitamin D were associated with lower odds of growth impairment, while gender and abnormal thyroid function were not significantly associated (Table 4).

**Table 4:** Generalized estimating equations (GEE) analysis of factors associated with overall growth impairment (stunting, underweight, and thinness) in internationally adopted children

Growth impairment (stunting, underweight and thinness)	OR	95% CI	*p*
Gender			
Male	1		
Female	0.89	0.69–1.43	0.374
Continent of origin			
Europe	1		
Asia	1.59	1.19–2.13	**0.002**
Africa	0.48	0.30–0.75	**0.002**
America	0.40	0.26–0.611	** < 0.001**
Age in years			
< 1 year	1.86	0.71–4.88	**0.021**
1–4 years	2.25	1.76–2.89	** < 0.001**
5–9 years	1		
Hb			
< 11 g/dl	1.62	1.01–2.59	0.043
≥ 11 g/dl	1		
Vitamin D			
< 20 ng/ml	0.71	0.54–0.93	**0.012**
≥ 20 ng/ml	1		
TSH [0,4–3,9 mIU/L]			
In range	1		
Not in range	0.98	0.71–1.36	0.918
Fetal-Alcohol Spectrum Disorder (FASD)			
Negative	1		
FASD	4.77	3.32–6.86	** < 0.001**

*OR* odds ratio, *CI* confidence interval, *Hb* hemoglobin, *TSH* thyroid-stimulating hormone, *TBC* tuberculosis, *FASD* fetal alcohol spectrum disorder, *FAS* fetal alcohol syndrome. *pFAS* partial fetal alcohol syndrome, *ARND* alcohol-related neurodevelopmental disorder, *ND-PAE* neurodevelopmental disorder associated with prenatal alcohol exposure

**Fig. 3:**
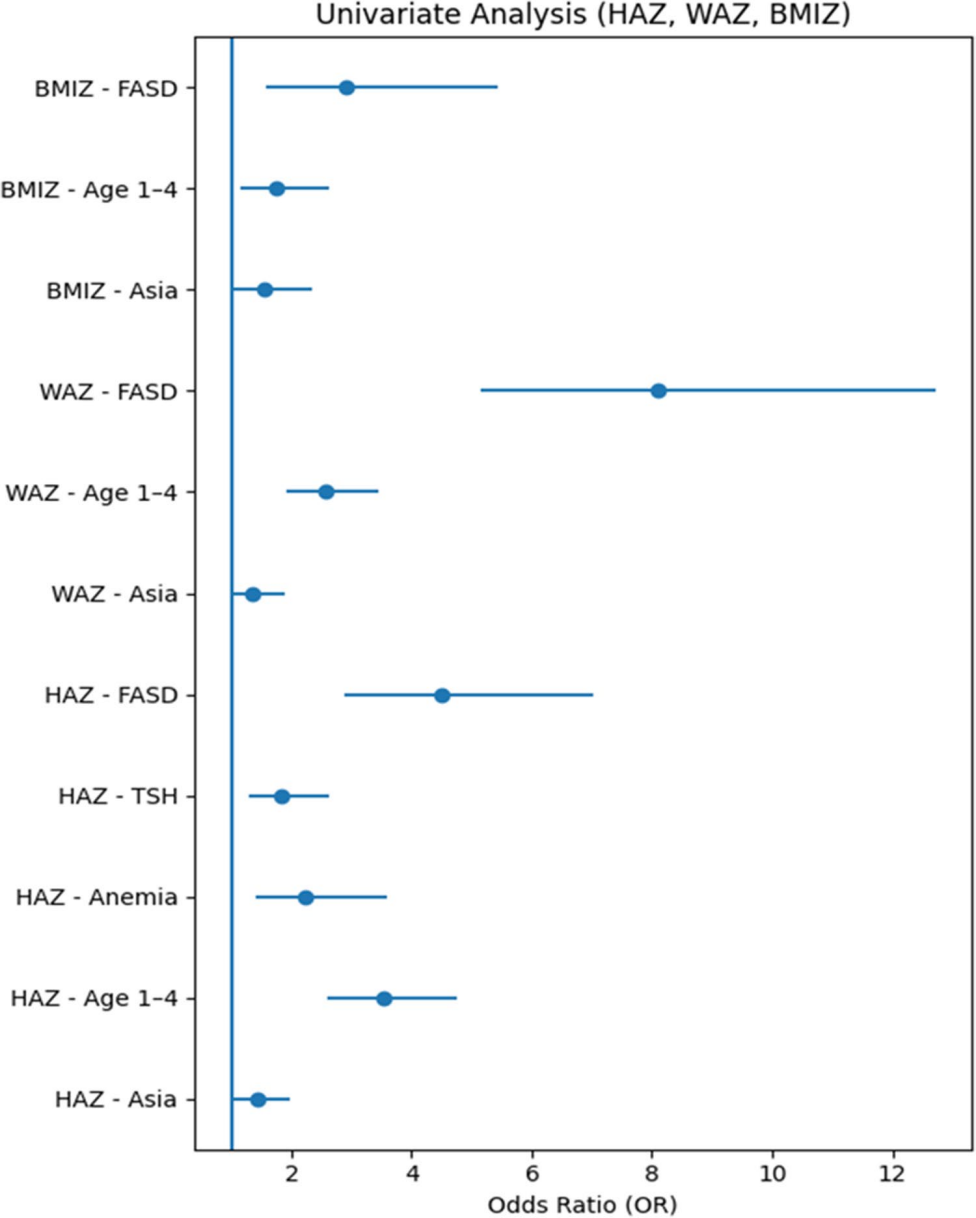
Univariate analysis of factors associated with stunting, underweight, and thinness in internationally adopted children. HAZ refers to height-for-age Z-score < − 2 standard deviations. WAZ, refers to weight-for-age Z-score < − 2 standard deviations; BMIZ refers to body mass index-for-age Z-score < − 2 standard deviations; OR, odds ratio; CI, confidence interval; FASD, fetal alcohol spectrum disorders; TSH, thyroid-stimulating hormone; Age 1–4 refers to children aged between 1 and 4 years

Univariate analyses were subsequently performed to identify variables most strongly associated with each specific outcome (thinness, stunting, and underweight). In univariate analyses, children originating from Asia, those aged 1–4 years, and those with suspected or confirmed FASD showed a higher risk of stunting, underweight, and thinness (Supplementary Tables S2–S4, Fig. 3). Moreover, stunting was significantly associated with anemia (OR 2.24, 95% CI 1.40–3.58) and with abnormal TSH levels (OR 1.83, 95% CI 1.28–2.63) (Supplementary material Table S2, Fig. 3).

### Laboratory alterations

The prevalence of eosinophilia did not significantly differ between children with and without stunting, thinness, or wasting (Tables 1–3).

Anemia was significantly more frequent among children with stunting (10% vs 5.9%, *p* = 0.01) (Table 1). Among the entire cohort of IAC, 110 children presented anemia at the first evaluation (5,6%), of whom 49 (2,5%) tested positive for a parasitic infection. Among 1845 non-anemic children (94.4%), 631 (32.3%) tested positive for a parasitic infection. Statistical analysis demonstrated a significantly higher proportion of parasitic infections among anemic children compared with non-anemic controls (*p* = 0.05). (Supplementary material Table S5.1).

Ferritin level was available only for 668 IAC. No statistically significant association was found between hypoferritinemia and stunting, underweight or thiness (Tables 1–3).

Abnormal TSH values were more common in the stunting group (18.3% vs 11.3%, *p* = 0.001) (Table 1), whereas no significant differences were found in the thinness or wasting groups (Tables 2–3).

Specifically, 662 (33,9%) IAC with available ferritin and TSH levels were analyzed. Of these 662 children, 117 (17,7%) had hypoferritinemia, including 24 (3,6%) with abnormal TSH levels and 93 (14%) with TSH values within the normal range. Among the 541 children (81.7%) with ferritin levels within the normal range, 68 (10.3%) showed abnormal TSH levels, whereas 473 (71.5%) had normal TSH values. Statistical analysis demonstrated a significant association between hypoferritinemia and abnormal TSH levels in the study population (*p* = 0.024) (Supplementary material, Table S5.1-S5.2) However, this association was not confirmed in multivariable logistic regression analysis, as neither hypoferritinemia, abnormal TSH levels, nor the interaction between the two variables showed a statistically significant independent association with stunting (Supplementary material, Table S5.1-S5.2). Although abnormal TSH levels were significantly associated with stunting in univariate analysis, this association was not confirmed in the interaction model restricted to children with complete ferritin and TSH data, likely due to reduced sample size and limited power for interaction testing.

Vitamin D deficiency (< 20 ng/mL) was not associated with stunting (30% vs 40.6%, *p* = 0.0022) and underweight (27.1% vs 39.4%, *p* = < 0.001). These results were confirmed by univariate analyses.

### Tuberculosis infection

The prevalence of tuberculosis infection did not significantly differ between children with and without stunting, underweight or thinness (Tables 1–3).

### Parasitic infections

No statistically significant differences were observed in the prevalence of parasitic infections between children with and without stunting, thinness, or underweight. In contrast, a significant association was found between parasitic infections and anemia, as reported above.

Among the 662 children with available ferritin levels and parasitic infection testing, 120 (18.1%) had low ferritin levels. Of these, 48 (7.3%) had a parasitic infection, whereas 72 (10.9%) did not. Among the 542 children (81.9%) with normal ferritin levels, 197 (29.8%) tested positive for a parasitic infection and 345 (52.1%) tested negative. No statistically significant association was found between low ferritin levels and parasitic infection (*p* = 0.45) (Supplementary material Table S5.1).

## Discussion

In this large cohort of IAC, we identified 12.2% of children with stunting, 12.7% of children with underweight, and 5.6% of children with thinness at the time of the first clinical evaluation. Independent risk factors for stunting, underweight and thinness included age at evaluation of 1–4 years, continent of origin, and fetal alcohol spectrum disorder. These findings are broadly consistent with the systematic review by Ivey et al., which analyzed 24 studies evaluating the nutritional status of IAC at arrival in the adoptive country. The prevalence of stunting ranged widely across studies, from approximately 12% to 39%, highlighting substantial heterogeneity in growth impairment among adopted populations. Notably, the prevalence of stunting reported in several of these studies was frequently higher than the estimates reported by the WHO for the general pediatric population in the same countries of origin [33]. Several studies have shown that older age at arrival is associated with a higher risk of malnutrition, likely reflecting longer exposure to institutional deprivation, inadequate nutrition, and untreated medical conditions [12, 33]. In contrast, in our cohort stunting and wasting were most prevalent among children aged 1–4 years, who accounted for approximately 60% of affected cases. This apparent discrepancy may be explained by the timing of evaluation: younger children are more likely to be assessed shortly after adoption, before substantial catch-up growth has occurred. Moreover, early childhood represents a period of heightened biological vulnerability, during which nutritional deprivation and infections may have a more pronounced impact on growth trajectories.

Consistent with global epidemiological patterns, children originating from Asia in our cohort showed the highest prevalence of stunting (18%), underweight (15.8%), and thinness (11%), reflecting the substantial burden of both chronic and acute malnutrition [34–36]. Interestingly, this finding differs from literature, which did not identify a consistent pattern of growth impairment according to country or region of origin among internationally adopted children; the marked heterogeneity of study populations and the limited availability of comparable data across countries may have obscured potential geographic differences [33]. In contrast, the large sample size of our cohort allowed a clearer evaluation of regional patterns of malnutrition among internationally adopted children.

In our study, the prevalence of FASD among IAC referred to our center for post-adoption screening was approximately 5%, consistent with previously reported European estimates [17]. According to current diagnostic guidelines [16, 17], prenatal alcohol exposure is a core component of FASD, while growth impairment is specifically associated with the diagnosis of FAS. We further explored the proportion of children with FASD who also met criteria for stunting (HAZ < − 2 SD), underweight (WAZ < − 2 SD), and thinness (BMIZ < − 2 SD). As expected, children with FASD were significantly associated with these conditions in both univariate and multivariate analyses. However, this association should be interpreted with caution, as growth impairment (both prenatal and postnatal) is intrinsically embedded in the diagnostic criteria for FAS, and prenatal alcohol exposure is itself linked to growth impairment. Therefore, while our findings confirm the overlap between FAS and stunting, underweight and thinness, FAS may not represent an independent risk factor for these conditions.

In a recent Italian cohort of IAC, anemia was confirmed as a frequent clinical condition at the time of the first post-adoption medical evaluation [37]. In low-resource settings, anemia commonly coexists with both stunting and wasting, and large pooled analyses have demonstrated that “stunting–anemia” and “wasting–anemia” comorbidities cluster among vulnerable children exposed to recurrent infections, chronic inflammation, and poor dietary quality [38, 39]. Our results demonstrated that anemia was more frequent at first evaluation in children with stunting compared with those without stunting (10% vs 5.9%, *p* = 0.01), as confirmed in univariate analyses. Helminth infections—particularly soil-transmitted helminths and schistosomiasis— contribute to chronic undernutrition, iron-deficiency anemia, inflammatory burden and impaired linear growth, thereby further compounding the risk of stunting [40, 41]. Our study demonstrated that anemia was significantly associated with parasitosis (*p* = 0.05) supporting the contribution of infectious and inflammatory mechanisms to hematologic impairment in this population [19].

Iron deficiency several months after arrival is associated with poorer developmental performance, with behavioral inattention and hyperactivity partially mediating cognitive outcomes [42]. These findings are consistent with broader evidence indicating that early-life iron deficiency can produce long-lasting alterations in brain structure and function, even after hematologic correction [43]. Moreover, iron deficiency may impair thyroid peroxidase (TPO) activity, as TPO is a heme-dependent enzyme that requires iron for its catalytic function [18]. Indeed, evaluating the relationship between iron status and thyroid function, a significant association emerged. Among children tested for both ferritin and TSH, abnormal TSH values were more frequent in those with hypoferritinemia. Abnormal TSH levels were significantly more common in stunted children as confirmed in univariate analysis. Undernutrition has been associated with reduced peripheral conversion of T4 to T3, alterations in TSH secretion, and a functional “low T3 syndrome,” reflecting metabolic adaptation to energy deprivation [44, 45]. In pediatric populations from resource-limited settings, thyroid dysfunction has been associated with impaired linear growth, and untreated hypothyroidism is a well-established cause of growth failure and delayed skeletal maturation [46].

Our results did not show an association between stunting, underweight, thinness, and vitamin D deficiency. These findings are in contrast with the existing literature [47–50]. However, our results may have been influenced by referral bias, as children referred to our center had typically already undergone an initial clinical assessment by their primary care pediatrician. This could explain why children presenting with evident signs of malnutrition and auxological criteria for stunting, wasting, and thinness may have received greater clinical attention, including earlier initiation of vitamin D supplementation.

This study has several limitations. First, its retrospective design may have introduced information bias and limited the availability of some clinical and laboratory variables. Second, although this represents one of the largest cohorts of internationally adopted children evaluated at arrival**,** the study was conducted in a single referral center, which may limit the generalizability of the findings to other adoption settings. In addition, information on pre-adoption living conditions, including duration and quality of institutional care, was often incomplete, preventing a more detailed evaluation of the impact of early-life environments on growth outcomes. Finally, the cross-sectional assessment at arrival did not allow evaluation of longitudinal catch-up growth after adoption.

## Conclusion

Growth impairment remains a substantial health concern among internationally adopted children at the time of their first medical evaluation. Our findings highlight the lasting impact of prenatal exposures, early-life deprivation, and adverse living conditions, particularly among children who experienced institutional care prior to adoption. Despite global efforts to reduce childhood malnutrition, a substantial proportion of these children still present with stunting and other indicators of undernutrition, suggesting that growth monitoring and nutritional support in institutional settings remain insufficient.

These results underscore the need for stronger health surveillance and nutritional care within child welfare institutions, where many children spend critical periods of early development. Improved monitoring of growth and early identification of nutritional deficiencies in these settings could help prevent chronic growth impairment before adoption. At the same time, internationally adopted children should undergo early and comprehensive medical screening after arrival, allowing prompt identification and management of growth deficits and associated risk factors.

## Supplementary Information

Below is the link to the electronic supplementary material.

Supplementary file1 (DOCX 18 KB)

Supplementary file2 (DOCX 21 KB)

Supplementary file3 (DOCX 21 KB)

Supplementary file4 (DOCX 20 KB)

Supplementary file5 (DOCX 15 KB)

## Data Availability

Data will be made available on reasonable request to the corresponding author.
