# Supplementary material for: Stunting, underweight and thinness in internationally adopted children: prevalence and associated factors in a large cohort study
Source: Eur J Pediatr. 2026 Jun 26;185(7):529. doi: 10.1007/s00431-026-07152-6 (PMC13303431; doi:10.1007/s00431-026-07152-6)
Supplement: Supplementary file 1 — Supplementary file1 (DOCX 18 KB) [file 431_2026_7152_MOESM1_ESM.docx]

**Supplementary material, Table S1.** Demographic and clinical characteristics of the study population

**Notes**

Hb, hemoglobin; TSH, thyroid-stimulating hormone; TBC, tuberculosis; FASD, fetal alcohol spectrum disorder; FAS, fetal alcohol syndrome; pFAS, partial fetal alcohol syndrome; ARND, alcohol-related neurodevelopmental disorder; ND-PAE, neurodevelopmental disorder associated with prenatal alcohol exposure.

| Characteristic of the study population | **Total of Internationally Adopted Children**  **(n=1955)** |
| --- | --- |
| Gender  Male  Female | 1186 (60.7%)  769 (39.3%) |
| Continent of origin  Europe  Asia  Africa  America  Unknown | 784 (40.1%)  418 (21.4%)  298 (15.2%)  447 (22.9%)  8 (0.4%) |
| Age in years  <1 year  1-4 years  5-9 years  10-14 years  15-18 years | 29 (1.5%)  660 (33.8%)  1021 (52.2%)  207 (10.6%)  38 (1.9%) |
| Days since arrival in Italy  <30 days  30-90 days  1-90 days  >90 days  Unknown | 234 (12.0%)  928 (47.5%)  1162 (59.4%)  724 (37.0%)  69 (3.5%) |
| Eosinophilia  No  Yes    Mild  Moderate  Severe | 1608 (82.3%)  347 (17.7%)  310 (15.9%)  36 (1.8%)  1 (0.1%) |
| Hb  <11 g/dl  ≥11 g/dl  Not performed | 126 (6.4%)  1821 (93.1%)  27 (1.4%) |
| Ferritin  <15ng/ml  15-300 ng/ml  Not performed | 120 (6.1%)  548 (28.0%)  1287 (65.8%) |
| Vitamin D  <20 ng/ml  ≥20 ng/ml  Not performed | 768 (39.3%)  1148 (58.7%)  39 (2.0%) |
| TSH range [0,4-3,9 mIU/L]  In range  Not in range  Not performed | 1661 (85.0%)  238 (12.2%)  56 (2.9%) |
| Coinfection parassities  No  Yes  Not performed | 1269 (64.9%)  680 (34.8%)  6 (0.3%) |
| TBC  No  Yes  Not performed | 1755 (89.8%)  196 (10.0%)  4 (0.2%) |
| Fetal-Alcohol Syndrome  Negative  FASD  FAS  pFAS, ARND,ND-PAE | 1860 (95.1%)  95 (4.9%)  26 (1.3%)  69 (3.5%) |
