# Supplementary material for: Stunting, underweight and thinness in internationally adopted children: prevalence and associated factors in a large cohort study
Source: Eur J Pediatr. 2026 Jun 26;185(7):529. doi: 10.1007/s00431-026-07152-6 (PMC13303431; doi:10.1007/s00431-026-07152-6)
Supplement: Supplementary file 2 — Supplementary file2 (DOCX 21 KB) [file 431_2026_7152_MOESM2_ESM.docx]

**Supplementary material Table S2.** Univariate analysis for factors associated with Stunting in International Adopted Children

**Notes:**

OR, odds ratio; CI, confidence interval; Hb, hemoglobin; TSH, thyroid-stimulating hormone; TBC, tuberculosis; FASD, fetal alcohol spectrum disorder; FAS, fetal alcohol syndrome; pFAS, partial fetal alcohol syndrome; ARND, alcohol-related neurodevelopmental disorder; ND-PAE, neurodevelopmental disorder associated with prenatal alcohol exposure.

|  | **Univariate analysis** |  |  |  |
| --- | --- | --- | --- | --- |
| **Study population characteristics** | **n/N** | **OR** | **95% CI** | ***p*** |
| Gender  Male  Female | 144/1186  95/769 | 1  1.02 | 0.77-1.34 | 0.889 |
| Continent of origin  Europe  Asia  Africa  America  Unknown | 105/784  76/418  25/298  31/447  2/8 | 1  1.43  0.59  0.48  2.15 | 1.04-1.98  0.37-0.93  0.32-0.73  0.43-10.8 | **0.028**  **0.025**  **<0.001**  0.351 |
| Age in years  <1 year  1-4 years  5-9 years  10-14 years  ≥15 years | 2/29  144/660  75/1021  17/207  1/38 | 0.93  3.52  1  1.13  0.34 | 0.22-4.00  2.61-4.74  0.65-1.95  0.046-2.51 | 0.927  **<0.001**  0.667  0.292 |
| Days since arrival in Italy  1-90 days  >90 days  Unknown | 155/1172  84/783 | 1  0.79 | 0.59-1.05 | 0.099 |
| Eosinophilia  No  Yes | 197/1608  42/347 | 1  0.99 | 0.69-1.41 | 0.939 |
| Hb  <11 g/dl  ≥11 g/dl  Not performed | 25/110  214/1845 | 2.24  1 | 1.40-3.58 | **0.001** |
| Ferritin  <15ng/ml  15-300 ng/ml  Not performed | 20/120  58/548  161/1287 | 1.69  1  1.21 | 0.97-2.93  0.88-1.66 | 0.062  0.24 |
| Vitamin D  <20 ng/ml  ≥20 ng/ml  Not performed | 72/768  160/1148  7/39 | 0.64  1  1.35 | 0.48-0.86  0.59-3.11 | **0.003**  0.47 |
| TSH [0,4-3,9 mIU/L]  In range  Not in range  Not performed | 183/1661  44/238  12/56 | 1  1.83  2.20 | 1.28-2.63  1.14-4.25 | **0.001**  **0.019** |
| Coinfection parassities  No  Yes  Not performed | 163/1269  75/680  1/6 | 1  0.84  1.36 | 0.63-1.13  0.16-11.69 | 0.244  0.781 |
| TBC  No  Yes  Not performed | 208/1755  30/196  1/4 | 1  1.34  2.48 | 0.89-2.04  0.26-23.94 | 0.162  0.433 |
| Fetal-Alcohol Spectrum Disorder  Negative  FASD  FAS  pFAS, ARND, ND-PAE | 205/1860  34/95  11/26  23/69 | 1  4.50  5.89  4.01 | 2.89-7.01  2.67 – 12.99  2.38 – 6.76 | **<0.001**  **<0.001**  **<0.001** |
