# Supplementary material for: Stunting, underweight and thinness in internationally adopted children: prevalence and associated factors in a large cohort study
Source: Eur J Pediatr. 2026 Jun 26;185(7):529. doi: 10.1007/s00431-026-07152-6 (PMC13303431; doi:10.1007/s00431-026-07152-6)
Supplement: Supplementary file 3 — Supplementary file3 (DOCX 21 KB) [file 431_2026_7152_MOESM3_ESM.docx]

**Supplementary material Table S3.** Univariate analysis for factors associated with underweight in International Adopted Children

**Notes:**

OR, odds ratio; CI, confidence interval; Hb, hemoglobin; TSH, thyroid-stimulating hormone; TBC, tuberculosis; FASD, fetal alcohol spectrum disorder; FAS, fetal alcohol syndrome; pFAS, partial fetal alcohol syndrome; ARND, alcohol-related neurodevelopmental disorder; ND-PAE, neurodevelopmental disorder associated with prenatal alcohol exposure.

|  | **Univariate analysis** |  |  |  |
| --- | --- | --- | --- | --- |
| **Study population characteristics** | **n/N** | **OR** | **95% CI** | ***p*** |
| Gender  Male  Female | 135/1043  82/667 | 1  0.94 | 0.70-1.26 | 0.694 |
| Continent of origin  Europe  Asia  Africa  America  Unknown | 107/680  78/385  17/268  13/370  2/7 | 1  1.36  0.36  0.20  2.14 | 0.98-1.88  0.21-0.62  0.11-0.35  0.41-11.18 | **0.062**  **<0.001**  **<0.001**  0.366 |
| Age in years  <1 year  1-4 years  5-9 years  10-14 years  ≥15 years | 5/29  126/660  86/1021  Not performed  Note performed | 2.27  2.57  1 | 0.84-6.09  1.91-3.44 | 0.105  **<0.001** |
| Days since arrival in Italy  1-90 days  >90 days | 146/1076  71/634 | 1  0.80 | 0.59-1.09 | 0.156 |
| Eosinophilia  No  Yes | 174/1406  43/304 | 1  1.17 | 0.81-1.67 | 0.401 |
| Hb  <11 g/dl  ≥11 g/dl | 17/105  200/1605 | 1.36  1 | 0.79-2.33 | 0.268 |
| Ferritin  <15ng/ml  15-300 ng/ml  Not performed | 18/106  54/476  145/1128 | 1.60  1  1.15 | 0.89-2.86  0.83-1.61 | 0.11  0.40 |
| Vitamin D  <20 ng/ml  ≥20 ng/ml  Not performed | 59/647  153/1029  5/34 | 0.57  1  0.99 | 0.42-0.79  0.38-2.59 | **0.0006**  0.98 |
| TSH range [0,4-3,9 mIU/l]  In range  Not in range  Not performed | 172/1438  32/220  13/52 | 1  1.25  2.45 | 0.83-1.88  1.28-4.69 | 0.28  **0.007** |
| Coinfection parassities  No  Yes  Not performed | 150/1107  66/597  1/6 | 1  0.79  1.28 | 0.58-1.08  0.15-11.00 | 0.140  0.824 |
| TBC  No  Yes  Not performed | 187/1547  28/160  2/3 | 1  1.54  14.55 | 1.00-2.38  1.31-161.19 | **0.051**  0.029 |
| Fetal-Alcohol Spectrum Disorder (FASD)  Negative  FASD  FAS  pFAS, ARND/ND-PAE | 176/1624  42/85  16/25  26 / 60 | 1  8.09  14.63  6.29 | 5.14-12.73  6.37 – 33.59  3.69 – 10.73 | **<0.001**  **<0.001**  **<0.001** |
