# Supplementary material for: Stunting, underweight and thinness in internationally adopted children: prevalence and associated factors in a large cohort study
Source: Eur J Pediatr. 2026 Jun 26;185(7):529. doi: 10.1007/s00431-026-07152-6 (PMC13303431; doi:10.1007/s00431-026-07152-6)
Supplement: Supplementary file 4 — Supplementary file4 (DOCX 20 KB) [file 431_2026_7152_MOESM4_ESM.docx]

**Supplementary material Table S4.** Univariate analysis for factors associated with Thinness in International Adopted Children

**Notes:**

OR, odds ratio; CI, confidence interval; Hb, hemoglobin; TSH, thyroid-stimulating hormone; TBC, tuberculosis; FASD, fetal alcohol spectrum disorder; FAS, fetal alcohol syndrome; pFAS, partial fetal alcohol syndrome; ARND, alcohol-related neurodevelopmental disorder; ND-PAE, neurodevelopmental disorder associated with prenatal alcohol exposure.

|  | **Univariate analysis** |  |  |  |
| --- | --- | --- | --- | --- |
| **Study population characteristics** | **n/N** | **OR** | **95% CI** | ***p*** |
| Gender  Male  Female | 70/1186  39/769 | 1  0.85 | 0.57-1.27 | 0.435 |
| Continent of origin  Europe  Asia  Africa  America | 53/784  42/418  10/298  4/447 | 1  1.54  0.48  0.12 | 1.01-2.35  0.24-0.95  0.04-0.35 | **0.046**  **0.036**  **<0.001** |
| Age in years  <1 year  1-4 years  5-9 years  10-14 years  ≥15 years | 4/29  49/660  45/1021  11/207 | 3.47  1.74  1  1.22 | 1.16-10.39  1.15-2.64  0.62-2.40 | **0.026**  **0.009**  0.569 |
| Days since arrival in Italy  1-90 days  >90 days | 60/1172  49/783 | 1  1.24 | 0.84-1.83 | 0.283 |
| Eosinophilia  No  Yes | 89/1608  20/347 | 1  1.04 | 0.63-1.72 | 0.867 |
| Hb  <11 g/dl  ≥11 g/dl | 6/110  103/1845 | 0.98  1 | 0.42-2.28 | 0.955 |
| Ferritin  <15ng/ml  15-300 ng/ml  Not performed | 9/120  21/548  79/1287 | 2.03  1  1.64 | 0.91-4.56  1.00-2.68 | 0.085  0.048 |
| Vitamin D  <20 ng/ml  ≥20 ng/ml  Not performed | 35/768  73/1148  1/39 | 0.70  1  0.39 | 0.46-1.06  0.05-2.86 | 0.095  0.353 |
| TSH [0,4-3,9 mIU/L]  In range  Not in range  Not performed | 89/1661  15/238  5/56 | 1  1.19  1.73 | 0.68-2.09  0.67-4.45 | 0.55  0.25 |
| Coinfection parassities  No  Yes | 74/1269  35/680 | 1  0.88 | 0.58-1.32 | 0.531 |
| TBC  No  Yes  Not performed | 97/1755  11/196  1/4 | 1  1.02  5.70 | 0.53-1.93  0.59-55.28 | 0.961  0.133 |
| Fetal-Alcohol Spectrum Disorder (FASD)  Negative  FASD, ARND/ND-PAE | 96/1860  13/95 | 1  2.91 | 1.57-5.42 | **0.001** |
