# Supplementary material for: Stunting, underweight and thinness in internationally adopted children: prevalence and associated factors in a large cohort study
Source: Eur J Pediatr. 2026 Jun 26;185(7):529. doi: 10.1007/s00431-026-07152-6 (PMC13303431; doi:10.1007/s00431-026-07152-6)
Supplement: Supplementary file 5 — Supplementary file5 (DOCX 15 KB) [file 431_2026_7152_MOESM5_ESM.docx]

|  | **Parasites infection** | **No Parasites infection** | **p-value** |
| --- | --- | --- | --- |
| **Anemia** | 49 | 61 | **P=0.026** |
| **No Anemia** | 631 | 1214 |  |

**Supplementary material Table S5.1.** Relationship between parasitic infection and anemia, low ferritin and thyroid dysfunction among IAC.

|  | **Parasites infection** | **No Parasites infection** | P=0.45 |
| --- | --- | --- | --- |
| **Low ferritin** | 48 | 72 |  |
| **Normal ferritin** | 197 | 345 |  |

|  | **Abnormal TSH** | **Normal TSH** | **P=0.024** |
| --- | --- | --- | --- |
| **Low ferritin** | 24 | 93 |  |
| **Normal ferritin** | 68 | 473 |  |

**Supplementary material Table S5.2** Logistic regression model assessing the interaction between low ferritin and abnormal thyroid-stimulating hormone levels among IAC.

|  | **OR** | **IC 95%** | **P-value** |
| --- | --- | --- | --- |
| **Low ferritin** | 1.26 | 0.39-4.11 | 0.70 |
| **Abnormal TSH** | 2.73 | 0.78-9.56 | 0.115 |
| **Low ferritin x Abnormal TSH** | 5.95 | 0.58-60.2 | 0.131 |

**Notes:**

OR, odds ratio; CI, confidence interval
